# Impact Response and Damage Tolerance of Hybrid Glass/Kevlar-Fibre Epoxy Structural Composites

**DOI:** 10.3390/polym13162591

**Published:** 2021-08-04

**Authors:** Vasudevan Alagumalai, Vigneshwaran Shanmugam, Navin Kumar Balasubramanian, Yoganandam Krishnamoorthy, Velmurugan Ganesan, Michael Försth, Gabriel Sas, Filippo Berto, Avishek Chanda, Oisik Das

**Affiliations:** 1 Department of Mechanical Engineering, Saveetha School of Engineering, SIMATS, Chennai 602105, India; navinkumarb.sse@saveetha.com; 2 Department of Mechanical Engineering, ARM College of Engineering and Technology, Kanchipuram 603209, India; yogakkm@gmail.com; 3 Department of Agricultural Engineering, Saveetha School of Engineering, SIMATS, Chennai 602105, India; velmurugang.sse@saveetha.com; 4 Structural and Fire Engineering Division, Department of Civil, Environmental and Natural Resources Engineering, Luleå University of Technology, 97187 Luleå, Sweden; michael.forsth@ltu.se (M.F.); gabriel.sas@ltu.se (G.S.); 5 Department of Mechanical Engineering, Norwegian University of Science and Technology, 13 7491 Trondheim, Norway; 6 Centre for Advanced Composite Materials, Department of Mechanical Engineering, The University of Auckland, Auckland 1142, New Zealand; avishek.chanda@auckland.ac.nz

**Keywords:** Kevlar, hybrid structural composites, hybridisation, impact loading, mechanical characterisation

## Abstract

The present study is aimed at investigating the effect of hybridisation on Kevlar/E-Glass based epoxy composite laminate structures. Composites with 3 mm thickness and 16 layers of fibre (14 layers of E-glass centred and 2 outer layers of Kevlar) were fabricated using compression moulding technique. The fibre orientation of the Kevlar layers had 3 variations (0, 45 and 60°), whereas the E-glass fibre layers were maintained at 0° orientation. Tensile, flexural, impact (Charpy and Izod), interlaminar shear strength and ballistic impact tests were conducted. The ballistic test was performed using a gas gun with spherical hard body projectiles at the projectile velocity of 170 m/s. The pre- and post-impact velocities of the projectiles were measured using a high-speed camera. The energy absorbed by the composite laminates was further reported during the ballistic test, and a computerised tomographic scan was used to analyse the impact damage. The composites with 45° fibre orientation of Kevlar fibres showed better tensile strength, flexural strength, Charpy impact strength, and energy absorption. The energy absorbed by the composites with 45° fibre orientation was 58.68 J, which was 14% and 22% higher than the 0° and 60° oriented composites.

## 1. Introduction

Polymer composite materials have been increasingly used in place of traditional materials in recent years due to their superior mechanical properties and ability to achieve desired properties through the selection of constituent materials. Over the last few decades, the use of high-performance composite materials has increased in structural applications that are prone to accidental dynamic loading. As a result, resistance to impact loading should be considered one of the primary requirements of high-pressure structural design [1]. The response of these materials to dynamic loading conditions has recently received a lot of attention from academia. Foreign objects with velocities of a few m/s to a few hundred m/s cause impact loading on structures, such as bird strikes, runway debris, maintenance tool drops, and projectiles. Impact events are commonly classified into three types based on their speed, projectile type, damage mechanism, and energy absorption behaviour, namely low-speed impact, high-speed impact, and hyper-velocity impact events [2,3]. When subjected to impact loading, composite materials exhibit complex failure modes, resulting in visible and invisible damage to object penetrations. As a result, the composite’s residual strength decreases. Understanding the response, energy absorption behaviour, and failure mechanism is thus a major concern that must be addressed. Extensive research is being conducted to investigate the energy absorption behaviour of polymer composite materials. Understanding dynamic loading conditions in composite materials is difficult because of the influence of various criteria such as composite material composition, projectile dimensions, velocities, target thickness, clamping methods, fibres and matrix, laminate thickness, layup sequence, geometry, and boundary conditions [4,5]. Polymer composites absorb energy during impact and undergo failure through different mechanisms, such as primary fibre failures, secondary fibre deformation, matrix cracks, delamination, shear plug formation, moving cone, and friction [6,7].

In general, Kevlar fibres are ductile and have high impact resistance, whereas carbon fibres have high strength, stiffness, and density [8,9]. However, these fibres are costly, whereas glass fibres are relatively inexpensive and have higher fracture strain and strength. The actual cost of glass-fibre reinforced thermosets is 62% higher than steel, compared to 76% for carbon-fibre reinforced thermosets. However, the exceptional weight reduction results in an overall weight savings of US$ 6.21 per pound in glass-fibre composites, which is only US$ 3.58 for carbon fibres [10]. Another structurally significant natural fibre is wood, which has immense applications in various industries and costs only US$ 0.10 per pound (lb) [11]. However, the strength of wood fibres is relatively lower, and the tensile strength to cost ratio can be calculated to be 134 MPa/$/lb for the glass-fibres compared to 180 MPa/$/lb for wood fibre reinforced composites [12]. Another advantage of glass-fibre composites is the reduced variability, which is significantly common in wood-based structures [13], and although wood is more sustainable, glass comes from sand and can be converted back to sand, which is also renewable and abundantly available, making it a sustainable product. On the other hand, Kevlar costs about US$ 13 per lb, making it significantly costly, although the tensile strength to cost ratio is quite high at 278.5 MPa/$/lb. Moreover, it should also be noted that Kevlar is 100% recyclable, although not very sustainable as it takes very long to decompose. Therefore, a successful hybridisation of glass-fibre and Kevlar-fibre layers can help to achieve an excellent product that gives a very high strength to cost ratio with increased applicability and considerably sustainable. Despite the fact that traditional polymer composite materials have a wide range of applications depending on their properties, certain drawbacks limit their usability. However, it is possible to retain the benefits of individual fibre properties through hybridisation. The hybridisation process of integrating high strain fibres with high stiffness fibres creates a new class of hybrid materials/structures with advanced performance properties. This hybrid concept is an appealing solution for bestowing the structure with high stiffness, toughness, and impact resistance at a low cost. Additionally, because the composite material properties depend on the constituents, it is possible to tailor the desired properties for specific applications [14]. Different types of hybrid composite structures can be fabricated depending on the constituent material mixture, resulting in desired mechanical properties. Following are some arrangements of hybrid composite structures:

- One material sandwiched by two layers of others;
- Alternate and mixed stacking sequences of two or more layers with different fibres;
- Reinforcement of two different short fibres/continuous fibres or short fibre with continuous fibre;
- Reinforcement of particles or other constituents to the aforementioned combinations.

Hybridisation brings effective results in the composites, which is evident by various research performed on polymer composites. Swolf et al. [15] developed hybrid carbon and glass fibre-based epoxy composites. This investigation reported a 15% increase in the composites’ failure strain due to hybridisation. Mehmet et al. [16] investigated the hybridisation effect of E-glass and Kevlar fibre-based epoxy composites. The composites were fabricated with different layering sequences. The hybrid composite with 10 layers of fibres (first two layers—glass fibre and next 8 layers—Kevlar fibre) showed a maximum tensile strength of 547 MPa. In contrast, for layered E-glass fibre epoxy composite, it was 441 MPa. Hosur et al. [17] investigated the low-velocity impact behaviour of the carbon/glass fibre epoxy composites. This investigation results proved the increase in the load carrying ability of the composites due to hybridisation. However, the composites stiffness was reduced. Chen et al. [18] investigated hybrid composites made of carbon/glass/basalt fibres. This investigation suggested the use of carbon fibre as a core layer for improving the impact resistance of the composites. From this investigation, it has been understood that selecting the optimum fibre as the core layer could increase the impact resistance ability of the composites. Pandya et al. [19] performed ballistic studies on plain-woven E-glass/epoxy, carbon/epoxy-based composites and their hybrids. The authors reported that hybridisation increases the ballistic limit, and placing the E-glass as the outer layer provides better performance compared to that obtained while placing carbon fibre as the outer layer. Gustin et al. [20] investigated the impact behaviour of carbon fibre and Kevlar-based hybrid composites. Adding one layer of Kevlar fibre to the carbon fibre composite produced effective results in the impact behaviour and maintained high stiffness for the composites. The investigation conducted by Valenca et al. [21] on Kevlar and glass fibre-based epoxy composite showed better mechanical strength, bending strength and impact energy owing to the effect of hybridisation. Naik et al. [22] reported a higher ballistic limit for E-glass fibre/epoxy than for T300 carbon fibre/epoxy and that E-Glass/epoxy absorbed energy through secondary yarn deformation and tensile failure of primary yarn, whereas T300 carbon fibre/epoxy had energy absorption through secondary yarn deformation and shear plugging. Results of this investigation [22] showed that a hybrid composite with glass fibre could be designed to exhibit a pronounced strain hardening behaviour with a strain capacity as high as 4%. From the aforementioned literature review, it can be inferred that reinforcement of E-glass into carbon fibre-based composite materials results in superior impact resistance.

Despite the steady progress of composite materials in structural applications, the low strength of glass fibres compared to carbon fibres and the low toughness and high cost of carbon, Kevlar and aramid fibres limit their applications, especially in dynamic loading conditions. Hybridisation of composite materials with different fibres is one of the best possible solutions to manifest the desired properties and expand composites’ applications in dynamic loading conditions. Numerous studies were conducted to fabricate various hybrid composite structures proving that hybridisation of reinforcement materials enhances mechanical and residual properties. It should also be noted that the mechanical properties and impact resistance of the hybrid composites depend on the integrated reinforcement materials; therefore, proper selection of materials for composite fabrication is critical. Moreover, the use of carbon fibre reinforced composite layers increases the amount of carbon footprint, which is attempted to be reduced by introducing a strong enough material made from glass-fibres and Kevlar reinforced composite layers. The current study is about investigating the mechanical and impact response of one such hybrid composite material having 16 layers of fibre (the outer Kevlar fibre layer and inner E-glass fibre layer). The hybrid epoxy composite laminates were fabricated through the compression moulding technique. Mechanical properties, namely tensile/flexural modulus, impact strength, shear strength and high-velocity impact strength were investigated. Projectile impacts were performed at a velocity of 170 m/s to determine the composite structures’ energy absorption capacity. Impact events were captured by a high-speed impact camera, and delamination processes were analysed by C-scan. The primary objective of this study was to evaluate the effect of hybridisation in impact response in terms of energy absorption behaviour and residual mechanical properties.

## 2. Materials and Methods

**Figure 1:**
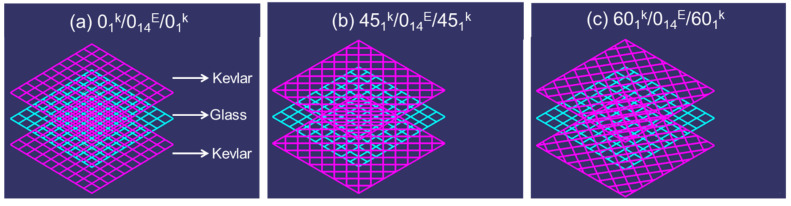
Schematics of the developed composite laminates.

For the present investigation, composite structures were fabricated using Kevlar and E-glass fibres. Both these fibres were purchased from the Marktech Composites Pvt Ltd., Bangalore, India, and the thermoset polymer epoxy-LY556 (CAS number 25068-38-6) with the preferred hardener (Aradur HY951) used was purchased from Huntsman, Pune, India. Three different kinds of hybrid composites were fabricated, each having a thickness of 3 mm with different ply orientations of Kevlar fibres (0°, 45° and 60°), whereas the glass fibres were orientation only at 0°. Composites were fabricated using the compression moulding process. During the fabrication, both the Kevlar and glass fibres were stacked into 16 layers, two layers of Kevlar fibres on top and bottom sides and fourteen layers of glass fibres between them. To maintain uniform thickness of 3 mm, 16 layers were preferred. Epoxy solution with the preferred hardener was mixed at the ratio of 10:1 as recommended by the supplier and was applied layer by layer and stacked together. Then the stacked layers were compressed at the pressure of 20 bar and temperature of 80 °C for 4 h. The prepared laminate size was 300 mm × 300 mm, which was then cut into 150 mm × 150 mm by band saw machine. The description of the composite designs is provided in Table 1 and Figure 1.

### 2.1. Experimental Setup Facility

#### 2.1.1. Tensile, Flexural, and Impact Strength

The tensile/flexural and impact properties of the composites were determined as per the ASTM D3039 and ASTM D790 protocols, respectively. The composites of size 300 × 25 × 3 mm were used for the tensile experimentation, whereas for the flexural test, the composite samples with the size 127 mm × 12.7 mm × 3 mm were used. For the Charpy (ASTM D6110) and Izod impact (ASTM D256) tests, composite samples with 127 mm × 12.7 mm × 3 mm and 60 mm × 12.7 mm × 3 mm were used, respectively. Five samples were tested for each testing method, and the average value was reported. The tensile and flexural properties of the fabricated samples were already reported in the previous articles [23,24] of the authors, and in this investigation, the results were compared.

#### 2.1.2. Interlaminar Shear Strength (ILSS)

The interlaminar shear strength test, according to ASTM D2344 standard, was performed on the composite specimens. The specimen of size 40 mm × 10 mm × 3 mm was used and tested under the three-point bending with 10 mm span. Five tests were again conducted under stroke-control at a crosshead speed of 2 mm/min using an electromechanical universal testing machine (Instron 3384) for each composite laminate. The interlaminar shear strength (ILSS) of the composites was found using Equation (1).

(1) $$\tau_{ILLS}=\frac{3F_{max}}{4wd}$$

where *w* and *d* are the width and depth, respectively, of the composite specimen used and Fmax is the maximum load.

#### 2.1.3. High-Velocity Impact Test

**Figure 2:**
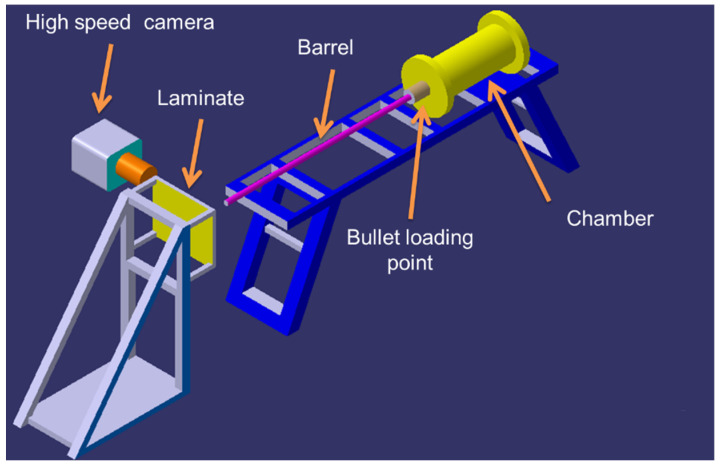
Schematic of experimental high velocity impact test setup.

**Figure 3:**
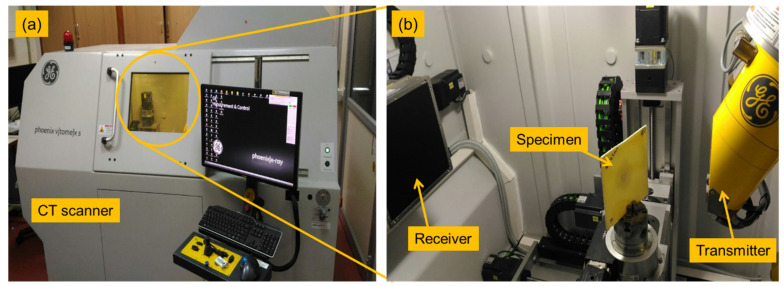
C-scan analysis instrument for high-velocity impact test. (**a**) CT scan machine used and (**b**) impact tested composite laminate subjected to analysis.

**Figure 4:**
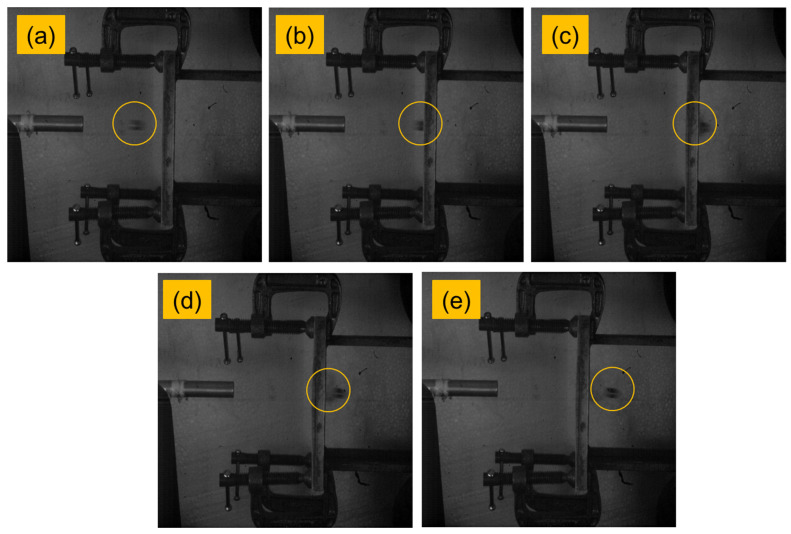
High-velocity camera images taken during the high-velocity impact test. (**a**) Projectile from barrel, (**b**) Projectile hitting the target, (**c**) Projectile piercing out of the target, (**d**,**e**) Projectile leaving the target.

The high-velocity impact test was carried out at the Laboratory of Aerospace Department, Indian Instituted of Technology—Madras, Chennai, India. In-house single-stage gas gun setup was used to perform the impact events where projectiles were driven by compressed air. A schematic diagram of the experimental setup is shown in Figure 2. High-speed camera, Phantom v11, was used to capture the events and compute the projectile’s velocities. The sample of size 150 mm × 150 mm × 3 mm was used for testing. Cylindrical projectiles made up of mild steel with a hemispherical nose were used for this study. Dimensions of the projectiles were 9.8 mm diameter, 10 mm length and 8 g weight. For each sample, one test was conducted, and the energy absorbed by the composites was found. The impact tested specimen was subjected to a computer tomography (CT)-scan for damage analysis. Figure 3 shows the CT-scan arrangement and setup. Figure 4 shows the high-velocity camera images taken during the high-velocity impact test.

## 3. Results and Discussion

### 3.1. Tensile, Flexural, Impact and ILSS Properties

The tensile properties of the laminates are shown in Table 2. The maximum strength of 232 MPa was noted for the composite with 45° fibre orientation, which was 2.5% and 5% higher than those of the composite with 0° and 60° fibre orientations, respectively. The failure strain was higher for the composite with 0° fibre orientation, followed by the 45° and 60° fibre orientation. A maximum failure strain of 1.3% was noted for the composite with 0° fibre orientation, which was 6% and 55% higher than the composite 45° and 60° fibre orientation. The tensile modulus of the composites was higher for the composite with 60° fibre orientation. Compared to the 0° and 45° fibre orientation, 60° fibre orientation composite showed 47% and 17% higher modulus value. These varying tensile properties are attributed to the difference in the fibre loading direction. These results clearly show the influence of fibre orientation against the tensile properties. Irrespective of the orientation, the tensile strength was observed to be significantly higher when compared to the established hybrid fibre-reinforced composite laminates in the present literature. Matykiewicz and Barczewski introduced [25] 6-layered flax (2 layers) and basalt (4 layers) hybrid composite laminates in epoxy resin mixed in varying concentration of silanised basalt powder, which had a maximum tensile strength of 205 MPa for 2.5 wt.% of basalt powder. The current material system also superseded that, irrespective of the fibre directions of the Kevlar layers (0° ~9.3%, 45° ~11.6%, 60° ~7.2%). The addition of Kevlar helped in increasing the tensile strength of the hybrid matrix from 200 MPa, reported by Zhang et al. [23]. Furthermore, the current samples also had greater strength (0° ~8.8%, 45° ~11.2%, 60° ~6.8%) when two carbon fibre-reinforced epoxy layups were hybridised with woven glass-fibre-reinforced layups [26].

Table 3 shows the flexural properties of the composites. The flexural strength value of the composites was higher than the tensile strength of the composites. This is because, during tensile loading, the composites experience stress throughout the surface area. However, in the case of flexural loading, the composites experience both tensile and compressive loading. In such a case, the composites experience stress in the small portion/area of the composite region, exactly on the top surface above the neutral axis. This reduces the chances of failure and increases the failure strain. For these reasons, the flexural strength of the composites was higher than that of the tensile strength. For the fabricated composites, the maximum flexural strength of 320 MPa was noted with 0° fibre orientation, which was 12% and 3% higher than the composite with 45° and 60° fibre orientation. The failure strain and modulus were also maximum for the 0° fibre orientation composite. The lowest failure strain and modulus were noted on the 60° fibre orientation composite. The composites with 0° fibre orientation was expected to have better properties in flexural primarily due to the orientation of the fibres, where all were oriented perpendicular to the bending axis. This resulted in higher bending stiffness, giving higher strengths to the hybrid laminates [27]. The overall flexural strength, again irrespective of the direction of the Kevlar layer, superseded quite a few of the fibre-reinforced structures outlined in the literature [25,26].

**Figure 5:**
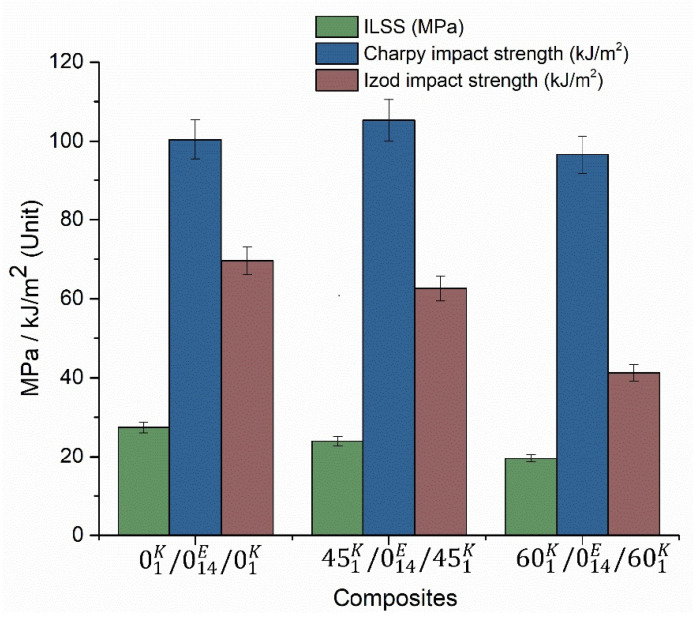
Impact strength and ILSS.

The impact strength result and ILSS results are shown in Figure 5. Huge variations have been observed between the Charpy and Izod impact strengths. In the Charpy impact strength case, maximum strength was noted for the 45° composite, whereas for the Izod test, the maximum strength was noted on the 0° composite. However, in both cases, the minimum strength was noted for the composite with 60° orientation. The maximum ILSS was noted for the composite with 0° fibre orientation, again as expected, which was 15% and 40% higher than those with the 45° and 60° fibre orientations, respectively. The rise in the tensile strength, flexural strain, flexural modulus and the Charpy impact strength, for the samples with the Kevlar layers being oriented at 45°, is quite interesting and can be attributed to the balanced orientation of the fibres, resulting in higher strengths. A similar result was found in the investigation of Retnam et al. [5], where composite with fibre orientation of 45° showed maximum impact strength of 87 kJ/m^2^. Studies on the creep analysis have been extensively performed regarding hybrid composite laminates with Kevlar, where the addition of Kevlar has resulted in increasing the creep behavior [28]. Moreover, the high strength of the Kevlar composite but substantially less strain-to-failure ratio, if complemented by a layer with high strain-to-failure ratio, can help in achieving a superior material system with potential for structural applications.

### 3.2. High-Velocity Impact Test Results

The high-velocity impact results of the composites are shown in Table 4 The results in the Table 4 was found through the use of the energy balance equation (Equation (2)).

(2) $$E_{impact}=E_{residual}+E_{composite\;plate}\;E_{composite\;plate}=E_{impact}-E_{residual}\;E_{CP}=E_{i}-E_{r}\;E_{impact}=E_{i}=\frac{1}{2}M_{P}\left[V_{i}^{2}\right]\;E_{residual}=E_{r}=\frac{1}{2}M_{P}\left[V_{r}^{2}\right]$$

The energy absorbed by the laminates (Equation (3)) is computed from the initial and residual velocities of the projectiles, which can be written as

(3) $$E_{CP}=\frac{1}{2}M_{P}\left[V_{i}^{2}-V_{r}^{2}\right]$$

where, *E_cp_* is the energy absorption of the composite laminate; *M_p_* is the projectile mass; and *V_i_* and *V_r_* are the initial and residual velocities of the projectile, respectively.

From the experiment results (Table 4), it can be seen that the energy absorption of the composite laminates changes with respect to the change in orientation of the fibre layer. The maximum energy absorption of 58.7 J was noted for the 45° orientation fibre laminate, whereas for 0° orientation fibre laminate, it was 44.8 J. A reduced energy absorption was noted on the 60° orientation fibre laminate. The reduction was 17% and 37% compared to the composite with 0° and 45° orientation fibre laminate, respectively. The energy absorbed by the composites is the function of the strength and failure strain of the composites. The variation in the energy absorption of the composites with the different laminates was mainly due to the variation in the composite modulus when changing the fibre orientation. The change in the modulus alters the strain rate of the laminates, and hence, the results of the fabricated composite laminates varied with respect to the orientation.

The residual velocity of the composites was lower for the laminate with 45° fibre orientation when compared to 0° and 60° orientations, showing 14% and 19% reduced residual velocity, respectively. High residual velocity was noted for the composite with 60° fibre orientation. In general, lower residual velocity indicates a better impact performance, and thus, the composite with outer layers having 45° fibre orientation showed better results than the other two composites. The main reason for the lowest residual velocity in the 45° orientation composites was the high resistance created by the composite against the shear strain and normal strain, which increased the failure strain rate. However, the 0° composite only withstood the normal strain, owing to the arrangement of the fibres in the loading direction. Moreover, the 60° layered composite was able to withstand shear and normal loads because loading along the off-axis angles was influenced by the coupling effects (i.e., axial and shear deformations). The uneven distribution of the load in 60° layered composite was the main reason for producing high residual velocity

**Figure 6:**
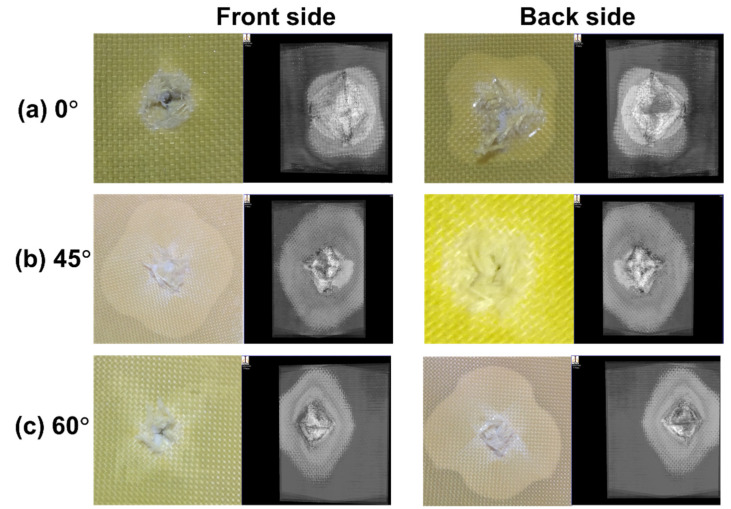
Damage on the front and backside of impact (photographic image and C-scan image).

The composite laminates were observed to experience greater damage in the back when compared to the front side, Figure 6. The damage in the front side of the composite laminate was akin to a hole having a diameter similar to the diameter of the impacted bullet. The delamination produced on the front side was also lower compared to the back of the laminate. The damaged areas of the 45° and 60° fibre-oriented composites were higher than the 0° orientation. This implies that the 45° composite showed high resistance against the projectile penetration that increased the damaged area, which was also evident for the increased energy absorption of the composites. The main reason was the ability of the 45° composites’ high failure strain rate. In general, higher energy absorption equates to a higher damage area. However, in the present case, when the energy absorption and damage area are compared between the 0° and 60° composites, the 0° composite has high energy absorption and lower damage area than the 60° composite. At 0° orientation, the laminate showed lower resistance for the projectile penetration because of poor resistance against shear strain, which can be observed through the crack formation perpendicular to the direction of the fibre. The 60° fibre orientation composite showed an uneven distribution of load due to the nonlinearity of axial and shear deformation that produced high delamination area. Similar results were found in the investigation of Murugan et al. [29].

**Figure 7:**
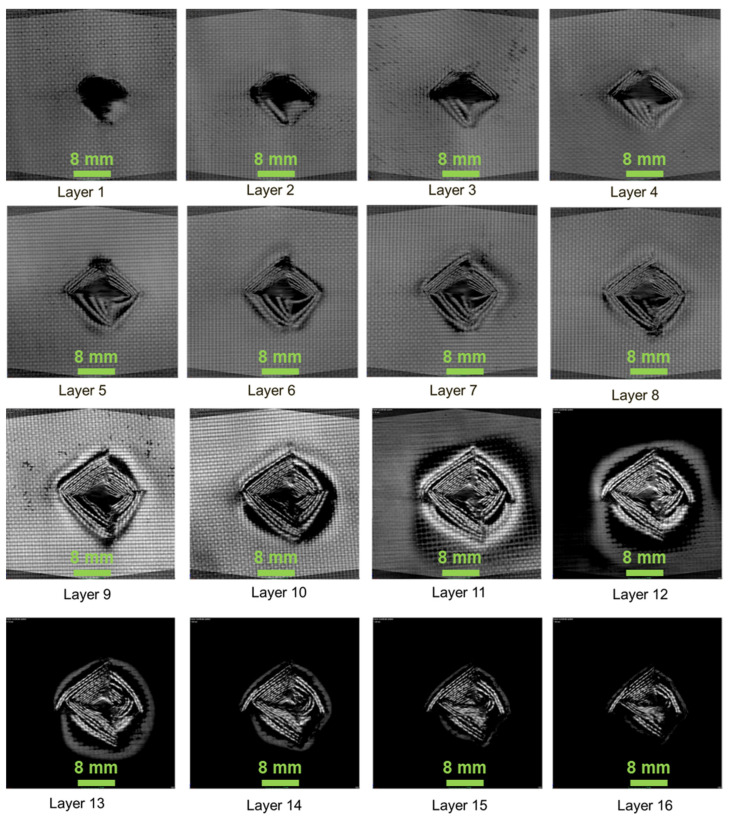
0_1_^k^/0_14_^E^/0_1_^k^—composite laminate failure on each layer.

**Figure 8:**
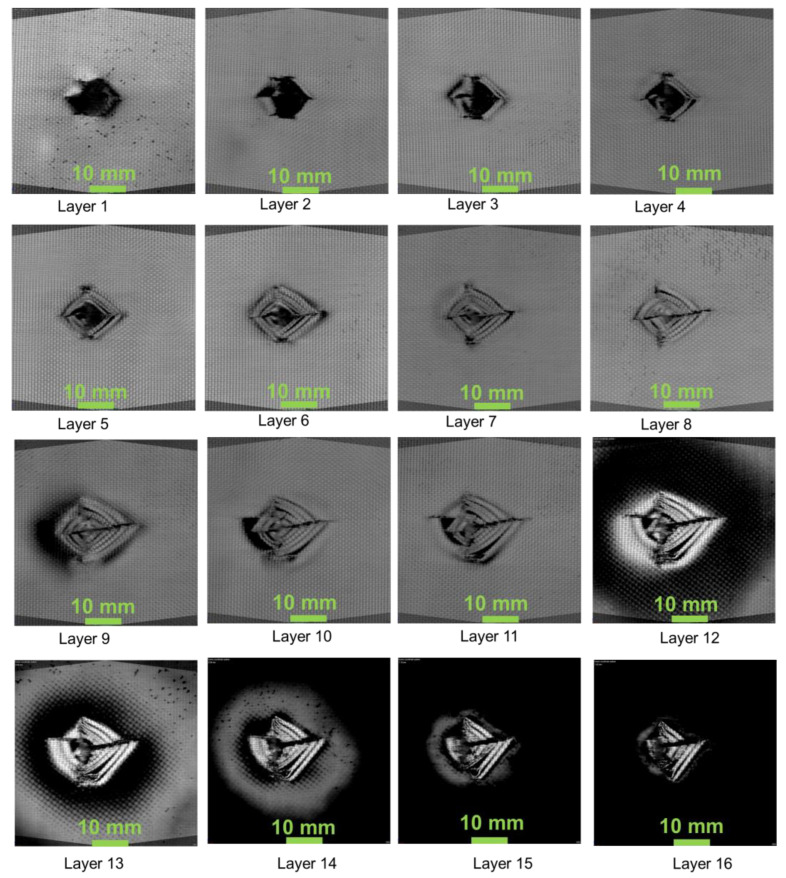
45_1_^k^/0_14_^E^/45_1_^k^—composite laminate failure on each layer.

**Figure 9:**
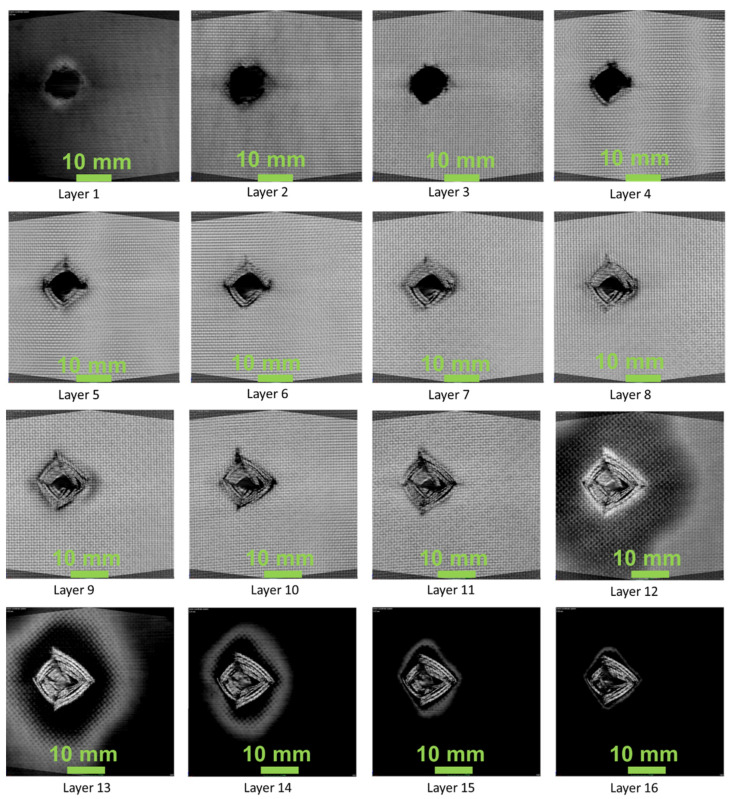
60_1_^k^/0_14_^E^/60_1_^k^—composite laminate failure on each layer.

Figure 7, Figure 8 and Figure 9 show the damage produced in the composites on each layer. From the figures, the layers at the bottom side of the laminate have shown compressive mode of failure, whereas the top layers experienced tensile failure. In layers 12, 13, 14, the damaged area produced due to the projectile impact is visible. The damage has been distributed more in the 45° compared to the 0° and 60° fibre orientations. It can be seen that energy absorbed by the 0° and 45° oriented composites was higher than that having 60° orientation. However, the damage produced was lower when compared to 0° oriented composite. This is mainly due to the fibre orientation, which in the case of 0° orientation showed lower impact damage owing to the concentrated damage created by the projectile. Thus, in the case of 0° orientation, the damaged area was less due to point impact. However, in the 45° and 60° laminated composites, the impact load was distributed around the impacted region, creating more damaged areas.

**Figure 10:**
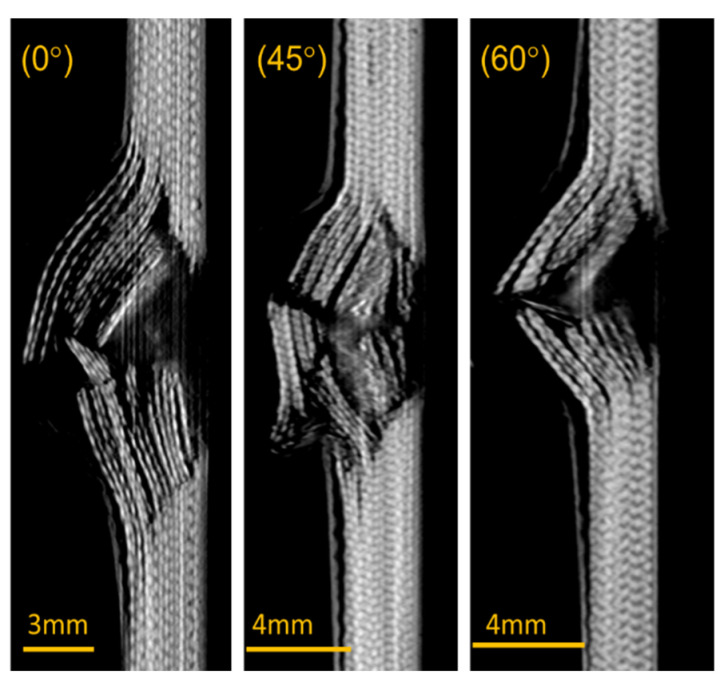
Cross-sectional view on the impacted region.

Figure 10 shows the side view of the X-ray computerised tomographic scan images of the composites. The composite with 45° fibre orientation had reduced rear plate bulge (cone radius) compared to the composite laminates with 0° and 45° fibre orientation. This explains the high energy absorption of the composites laminate with 45° fibre orientation. The 0° composite has ‘through’ hole due to impact of the projectile at a concentrated load. Murugan et al. [29] reported that the higher the cone radius, the lower is the energy absorption. As explained, the 60° orientation had a high cone radius and lower energy absorption compared to the other two fibre orientations.

## 4. Conclusions

Hybrid epoxy composite with 0°, 45° and 60° orientation Kevlar fibre and 0° orientation E-glass fibre was successfully fabricated and investigated for the tensile, flexural, impact, interlaminar shear strength and high-velocity impact properties. Based on the experiments, the following results are obtained:

- The maximum tensile strength (ca. 232 MPa) of the composites was noted on the composite with 45° fibre orientation (ca. 2.6% and ca. 4.7% higher than 0° and 45° fibre orientations, respectively) and maximum failure strain (ca. 1.3%) was obtained on the 0° fibre orientated composite. The maximum tensile modulus of ca. 26 GPa was noted on the 60° fibre orientated composite.
- The flexural properties of the composite were high for the 0° fibre orientated composite when compared to 45° (ca. 10.9%) and 60° (ca. 2.5%) fibre orientated composites. The maximum strength, failure strain and modulus were ca. 321 MPa. 18% and 12 GPa, respectively.
- Compared to the Izod impact strength, Charpy impact strength showed the maximum strength. The maximum Charpy impact strength (ca. 105 kJ/m^2^) was noted for 45° fibre-oriented composite, whereas the maximum Izod impact strength (ca. 70 kJ/m^2^) was noted for 0° fibre-oriented composite.
- The maximum interlaminar shear strength of ca. 27 MPa was observed on the 0° fibre-oriented composite and minimum (ca. 20 MPa) was noted on the 60° fibre-oriented composite.
- In the high-velocity impact test, 45° fibre-oriented composite showed better results. The energy absorbed by the composite was ca. 58 J (ca. 23.7% > 0° orientation and ca. 36.8% > 60° orientation, of the Kevlar layers) and the velocity drop was ca. 62 m/s, which was again ca. 28.3% and ca. 42.2% higher than the samples having 0° and 45° fibre orientations, respectively. The residual velocity of the 45° fibre-oriented composite was ca. 108 m/s, being ca. 13.9% and ca. 19.5% lower than those oriented at 0° and 60°, respectively.

The fabricated composites were found to be a better candidature for the structural applications from the experimental results. Overall, the composite with 45° fibre orientation performance was notable compared to other composites. Based on the mechanical and high-velocity impact test results, the composites having 45° fibre orientation of the Kevlar layers can be the best choice for utilisation in structural and other applications, such as building, automobile, marine and aerospace industries, where high-velocity impact is common.

## Figures and Tables

**Table 1:** Composite laminate formulation.

Specimen Name	Top Layer		Middle Layers		Bottom Layers		Ply Orientation	Total No. of Ply	Total Thickness, mm
	Fibre	No. of Ply	Fibre	No. of Ply	Fibre	No. of Ply			
[0_1_^k^/0_14_^E^/0_1_^k^]	Kevlar	1	E- Glass	14	Kevlar	1	0/0/0	16	3
[45_1_^k^/0_14_^E^/45_1_^k^]	Kevlar	1	E- Glass	14	Kevlar	1	45/0/45	16	3
[60_1_^k^/0_14_^E^/60_1_^k^]	Kevlar	1	E- Glass	14	Kevlar	1	60/0/60	16	3

**Table 2:** Tensile properties for hybrid composites.

Material	Tensile Strength (MPa)	Failure Strain (%)	Tensile Modulus (GPa)
[0_1_^k^/0_14_^E^/0_1_^k^]	226 * ± 2.3	1.3 * ± 0.1	17 * ± 0.5
[45_1_^k^/0_14_^E^/45_1_^k^]	232 ** ± 3.0	1.2 ** ± 0.12	21 ± 1.35
[60_1_^k^/0_14_^E^/60_1_^k^]	221 ** ± 1.3	0.8 ** ± 0.09	26 ± 0.3

* Data taken from Ref [23], ** Data taken from Ref [24].

**Table 3:** Three-point bending properties for E-glass/epoxy and different hybrid composites.

Material	Flexural Strength (MPa)	Failure Strain (%)	Flexural Modulus (GPa)
[0_1_^k^/0_14_^E^/0_1_^k^]	321 * ± 1.13	18 * ± 0.9	12 * ± 0.5
[45_1_^k^/0_14_^E^/45_1_^k^]	286 ** ± 2.6	15 ** ± 1.06	12 ± 1.2
[60_1_^k^/0_14_^E^/60_1_^k^]	313 ** ± 1.6	12.6 ** ± 1.5	10.5 ± 0.9

* Data taken from Ref [19], ** Data taken from Ref [20].

**Table 4:** Energy absorption of composite laminates.

Sample	Initial Velocity (m/s) *V_i_*	Residual Velocity (m/s) *V_r_*	Velocity Drop (m/s) *V_d_*	Energy Absorption Composite (J) *E_cp_*
[0_1_^k^/0_14_^E^/0_1_^k^]	170	125.3	44.5	44.8
[45_1_^k^/0_14_^E^/45_1_^k^]	170	107.9	62.1	58.7
[60_1_^k^/0_14_^E^/60_1_^k^]	170	134.08	35.9	37.1

## Data Availability

The data that support the findings of this study are available from the corresponding author upon reasonable request.
